# Prevalence and predictors of smoking cessation among smokers receiving smoking cessation intervention in primary care in Qatar: a 6-month follow-up study

**DOI:** 10.3389/fpubh.2023.1166016

**Published:** 2023-05-19

**Authors:** Ayman Al-Dahshan, Hissa Al Muraikhi, Sarah Musa, Anwar Joudeh, Wadha Al Baker, Nagah Selim, Iheb Bougmiza

**Affiliations:** ^1^Department of Medical Education, Hamad Medical Corporation, Doha, Qatar; ^2^Department of Preventative Health, Primary Health Care Corporation, Doha, Qatar; ^3^Department of Internal Medicine, Hamad Medical Corporation, Doha, Qatar; ^4^Department of Community Medicine, Primary Health Care Corporation, Doha, Qatar; ^5^Department of Public Health and Preventive Medicine, Cairo University, Giza, Egypt

**Keywords:** quit rate, smoking cessation, predictors, primary care, Qatar

## Abstract

**Objective:**

To estimate the rate and predictors of smoking cessation in smokers attending smoking cessation clinics in primary care settings in Qatar.

**Methods:**

A cross-sectional study was conducted among 759 smokers who had attended any of the 10 smoking cessation clinics in primary health care centers from January 2019 to June 2020. The sociodemographic, clinical, and smoking-related variables were assessed. Tailored behavioral and pharmacotherapy were delivered, and patients were interviewed at 6 months to estimate the 30-day point prevalence abstinence. To identify independent factors associated with smoking cessation, a multivariable logistic regression analysis was performed.

**Results:**

The mean age of participants was 40.6 (±11.3), majority being married, Arab and employed, and having a tertiary education. Almost half of the smokers (48.7%) received varenicline alone, 42.6% received NRT, and 31.8% received a combination of both. The selection of drug therapy was based on preferences, experiences, and history of previously encountered adverse effects. The overall 30-day quit rate at 6 months follow-up was 32.4%. About three-quarters (72.5%) of participants had at least one quit attempt and 12.5% had 3 or more attempts. Later age at smoking initiation, lower cigarette consumption at baseline, lower CO concentration at baseline, use of smoking cessation pharmacotherapy, having made fewer quit attempts and non-exposure to secondhand smoke among friends were identified as significant predictors of successful quitting at 6 months.

**Conclusion:**

The 30-day quit rate at 6 months follow-up (32.4%) is comparable to the worldwide figure. However, further efforts should be made to plan cost-effective tobacco dependence treatment taking into account predictors and at-risk groups.

## Introduction

Tobacco smoking remains a growing public health threat and a leading cause of preventable premature mortality worldwide. Globally, an estimated 6 million deaths are attributed to smoking annually and if the current trend continues, by 2030 smoking will lead to an estimated 8 million deaths per year with more than 80% taking place in low-middle-income economies (1–3). Efforts to combat tobacco smoking epidemics are ongoing with a projected decline of smoking rate by 2025 (4). However, rapid population growth and pressure from the global tobacco industry have largely contributed to an increase in the total number of smokers (5, 6) with more than billion smokers worldwide (7). Regions witnessing the greatest surge in tobacco use include Eastern Mediterranean (Jordan, Oman, Lebanon, Egypt), Africa (Congo) and Europe (Moldova) (4).

The World Health Organization (WHO) has made a vast advancement in saving millions of lives by ensuring the implementation of the Framework Convention on Tobacco Control. The framework calls for a reduction in tobacco smoking prevalence by 30% by 2025 (8). This goal was further integrated within WHO’s global non-communicable disease monitoring framework (9) as well as the Sustainable Development Goals of the United Nations (10).

Tobacco dependence is not easy to quit. The average cessation rate is estimated to be 5% for those who try quitting on their own, (11) and increases by 16% for those who obtain prescribed smoking cessation medications (12). Evidence indicates that while half of smokers have intentions to quit and one-third has made at least three attempts in the preceding year, less than 50% were successful in quitting before the age of 60 (13).

Smoking cessation at any age is associated with substantial benefits for smokers, communities, and healthcare costs. The risk of premature death declined by 90% when quitting occurs before the age of 40 (14). Treating tobacco dependence is shown to be more cost-effective when compared to many common disease prevention interventions such as statin, anti-hypertensive drugs, and cervical cancer screening (15). Investing 1.68 US dollars *per capita* annually in evidence-based anti-tobacco interventions such as free quit lines and supportive consultations could help 152 million tobacco users to quit successfully (16).

Earlier literature has revealed significant associations between cigarette smoking and certain sociodemographic characteristics. For example, a cross-sectional study in Greece found that male gender, younger age and higher educational level were factors associated with smoking (17). In contrast, analysis from a nationally representative survey in China found that middle-aged males and lower educational level were associated with smoking (18). In addition, certain genetic factors are shown to be associated with a higher risk of early age smoking initiation and the risk of relapsing after smoking cessation (19). Moreover, several motivational, behavioral and knowledge factors were correlated with the intention to quit smoking. While having past quit attempts as well as lower amount of smoked tobacco and expected health benefits were positively linked to the intention to quit smoking, other measures such as being ambivalent toward enjoying smoking, having no regret on starting smoking, high perceived vulnerability and low self-efficacy were adversely associated with intention to quit smoking (20–23).

Behavioral and pharmacological interventions including individual/group therapy, nicotine replacement therapy, varenicline and bupropion were demonstrated to be effective in supporting smoking cessation (24–26). Previous studies have also provided evidence of structured hospital smoking cessation services being a contributing factor in increasing the rate of successful quitting (27, 28). Data suggests that assisted interventions, either single or combined, have increased the rate of successfully quitting smoking at 6 months ranging 35–55% (29).

In Qatar, the prevalence rate of tobacco smoking among the adult population has witnessed a drop from 36.7% in 2000 to 25.2% in 2019 (30). Despite that, smoking rates in Qatar remain high especially among vulnerable groups as indicated by the 2013 Global Adult Tobacco Survey (12% among aged 15 years and older), calling for extra sustainable and collaborative efforts to curtail smoking initiation and eliminate barriers that hamper cessation (31). Several effective tobacco control measures were adopted by Qatar including tobacco taxes, designation of smoke-free areas, anti-smoking health awareness, and expansion of free or subsidized tobacco dependence treatment services such as smoking cessation clinics at Primary Health Care (PHC) centers (30). These clinics are operated by qualified and trained physicians who provide clients with behavioral counseling for smoking cessation as well as pharmacotherapy which includes Varenicline and Nicotine Replacement Therapy (NRT)- short-acting (lozenges) or long-acting (patches).

Studies on tobacco control conducted in Arab countries are mainly descriptive and focus on the prevalence and type of tobacco smoking (32). Studies evaluating the effectiveness of cessation interventions in Arab countries are scarce. For example, a cross-sectional study in Bahrain among 194 smokers who attended two outpatient cessation clinics found that the 6-month quit rate was 37.6% (33). Successful quitting was associated with being unemployed, having higher counseling sessions, and a higher number of quit attempts (33). Another study in an outpatient cessation center in Lebanon among 156 smokers evaluated the effectiveness of a 3-month cessation program. The authors found that smokers with a higher number of smoked packs per year were less likely to achieve successful tobacco quitting, while compliance with the treatment increased the odds of quitting success by 7.6 times (34). In addition, a study in Saudi Arabia found that the 3-month quit rate among smokers who utilized cessation services was 26.0%. Reasons for successful quitting included receiving counselling services, NRT, and social/family support (35).

These studies provided baseline descriptive data on the effectiveness of cessation services in the Arab region. However, most of the studies involved a convenient small sample of smokers, and no study has assessed the predictors of successful smoking cessation. A recent review indicated that tobacco control in the Eastern Mediterranean Region is witnessing the smallest decline when compared to other regions, making the WHO’s 2025 target unachievable (32). The lack of research on cessation interventions was cited as one of the barriers. Therefore, assessing cessation clinics with a nationally representative sample will provide evidence-based guidance for planning cost-effective cessation interventions and maximizing quit rates. In this study, we aimed to (i) estimate the 30-day smoking quit rate at 6 months of the initial visit, and (ii) identify predictors of successful smoking quitting among smokers attending smoking cessation clinics in primary care in Qatar.

## Materials and methods

### Study design and setting

An analytical prospective cross-sectional design was conducted at all 10 smoking cessation clinics in PHC centers in Qatar from January 1st, 2019 to June 30th, 2020. The recruitment of participants started on January 1st and ended on December 31st 2019, while the follow-up interviews took place between June 1st, 2019 and June 30th, 2020. These clinics are distributed across the three geographic regions of the country (Northern, Southern, Western). Therefore, a proportionate stratified sampling technique was used to estimate a representative sample conformed to the population densities (Table 1). Smokers can access smoking cessation clinics either through self-referral (via calling a hotline) or family and community medicine physician referrals. The clinics provide free or highly subsidized quit-smoking services for all adult smokers who reside in Qatar.

**Table 1:** Distribution of respondents according to the smoking cessation clinics in Qatar 2020 (Proportionate stratified sample) (*N* = 759).

Health center	Population	
	Frequency	Percent (%)
Gharrafat Al Rayyan	171	22.5
Abu Baker Al Siddiq	118	15.5
Rawdat Al Khail	115	15.2
Al Wakra	95	12.5
Mesaimeer	71	9.4
Leabaib	66	8.7
Qatar University	52	6.9
Omar Bin Al Khattab	41	5.4
Al Ruwais	18	2.4
Al Daayen	12	1.6

### Smoking cessation program

Services at smoking cessation clinics are based on two parts: (1) behavioral by helping the smoker to stay away from the habit, increasingly important at the initial treatment stage as well as promoting the adoption of a healthy lifestyle, and (2) pharmacotherapy by prescribing medications to help smokers with nicotine dependency.

During the initial visit to the smoking cessation clinics, motivated smokers received a brief introduction of the smoking-cessation program, and completed baseline questionnaires related to their tobacco use and smoking history. Exhaled CO level was measured for every participant during the initial visit.

At this initial as well as each subsequent visit, smokers were provided with tailored behavioral counseling delivered by Tobacco Treatment Specialists. Behavioral counselling could involve motivational interviewing, cognitive behavioral therapy and positive psychology as appropriate. It takes into account patients’ behaviors, values, and beliefs, and offers patients information, guidance and counseling regarding lifestyle-related behaviors, the role of drug therapy as well as the need for social support for achieving successful abstinence.

Drug therapy options included varenicline, bupropion SR, NRT (nicotine lozenges and nicotine patches), or combination therapy. The selection of drug therapy was based on several factors: clinicians’ familiarity with the medications; contraindications for selected patients; patient preferences; and previous patient experience with a specific pharmacotherapy (positive or negative); after shared clinical decision-making. Drug doses were adjusted according to manufacturer’s product information. According to each patient’s needs, the duration of the drug therapy was determined; however, a minimum of 12 weeks duration was recommended.

A personalized quit plan including the target quit date was discussed with each patient. Follow-up, through face-to-face or phone consultations, was delivered on or around the quit date and monthly thereafter, or as per need or request from the patient. Both, behavioral and drug therapies including tolerability and effectiveness were continuously monitored according to the patient’s progress throughout the program.

### Study population

The study population was adult tobacco smokers attending any of the 10 smoking cessation clinics for the intention to quit smoking within the duration of the study period, and who met the following inclusion criteria: (i) able to understand and speak Arabic or English, (ii) were aged 18 years or above, and (iii) had an active mobile or land phone number. Participants with a life-threatening illness such as cancer or cognitive dysfunction were excluded.

### Sample size

The estimated sample size was 768 smokers based on 5% absolute precision, 95% confidence interval, a hypothesis that 50% of smokers who attend the smoking cessation clinics succeed in quitting smoking, and a non-response rate of 20%. “Non-response” was defined as the failure to reach the potential participant after three consecutive daily calls.

### Measures and variables

Participants were defined as smokers if they had been smoking continuously or accumulatively for 6 months or more in their lifetime. A regular smoker is referred to as one who smoked one or more cigarettes per day for a period of 6 months or more. Any attempts to quit by smokers within the past 6 months were identified as an “attempt to quit” smoking. Successful quitters were defined as smokers who reported being abstinent for at least 30 days at the six-month follow-up interview.

The study outcome was self-reported 30-day point-prevalence abstinence from cigarette smoking, which is defined as not smoking even a puff for the past 30 days when questioned at 6 months following the quit date. Those who reported 30-day point prevalence abstinence were further evaluated for 6 months abstinence being as smoking free even a puff in the past 6 months.

### Data extraction

- Sociodemographic and clinical characteristics recorded at baseline included age, sex, educational level, employment status, marital status, body mass index (BMI), presence of comorbidities and type of comorbidities. Data were retrieved from patients’ electronic medical records (EMRs).
- Smoking-related characteristics recorded at baseline (first visit) included age of starting smoking, duration of smoking in years, number of daily smoked cigarettes, and exhalatory carbon monoxide (CO) concentration. Exhalatory CO test value (ppm) was categorized into the following: 0–9 for non-smoking; 10–19 is light smoking; 20–29 for moderate smoking; 30 or more for heavy smoking. Values were collected and measured at the smoking cessation clinic by a trained doctor or nurse.
- Smoking-related characteristics recorded at the six-month follow-up included type of therapy for smoking cessation, medication adherence, exposure to secondhand smoke among friends, exposure to smoking at home and the number of smokers at the same home. Data were collected via a standardized telephone assessment by trained investigators 6 months after the quit date. Monitoring of medication adherence was assessed also through attendance at scheduled visits.
- Smoking cessation status and abstinence time were recorded at the six-month follow-up as the duration between the day smoking was stopped to the day of assessment. Data were collected via a standardized telephone assessment by trained investigators 6 months following the quit date.

### Instruments

The data collection was obtained through a checklist designed to collect relevant data from participants’ EMRs of smoking cessation clinics’ encounters as well as through a structured questionnaire. To ensure the face and content validity of the used tool, an exhaustive literature review was undertaken by an expert committee made of Tobacco Treatment Specialists, Public Health Specialists, and Community and Preventive Medicine consultants. The questionnaire was in English and Arabic (the main communication languages of residents in Qatar). Cronbach’s alpha coefficient was 0.839, indicative of acceptable reliability. A pilot trial was undertaken with a convenience sample of (*n* = 30) smokers to appraise the questionnaire for relevance, clarity, and time taken to complete it. The data generated was omitted from the final analysis.

### Statistical analysis

Data were analyzed using *IBM SPSS Statistics for Windows* (version 23, IBM Corp., Armonk, N.Y., United States). Descriptive statistics in the form of frequencies and percentages (categorical variables), and means ± standard deviation or median ± interquartile range (IQR), depending on data distribution (continuous variables) were utilized. The chi-square was used to assess the statistical significance between categorical variables. In addition, a multivariable logistic regression model was built to examine the associations between successful smoking cessation at six-month follow-up and the independent variables. The Hosmer-Lemeshow’s test was used to construct the final regression models. Adjusted odds ratios (ORs) with their corresponding 95% confidence intervals were reported. Statistical significance was considered at *p* ≤ 0.05.

## Results

### Characteristics of participants

Of the 919 participants enrolled in the smoking cessation program, 160 participants (17.4%) were lost to follow-up while 759 (82.6%) were included in this study. The sociodemographic and clinical characteristics of the study participants are shown in Table 2. The mean age was 40.6 (±11.3) with the majority being males (95.0%), married (85.4%) and Arabs having the nationality of one of the Arab League States such as Egyptian, Qatari, Syrian, Jordanian, Palestinian, Lebanese, Tunisian, Sudanese, Yemeni, Saudi, Iraqi, Bahraini, Moroccan, and Omani (86.8%). More than two-thirds (69.6%) of the sample had a tertiary level of education and most participants (86.7%) were employed. The mean Body Mass Index (BMI) of the participants was 29.1 (±5.3) and about one-third (31.5%) had one or more chronic diseases.

**Table 2:** Socio-demographic and clinical characteristics of smokers recorded at first visit (baseline) to smoking cessation clinics in Qatar (*N* = 759).

Variable	Frequency	Percent (%)
Age (years)		
18–24	57	7.5
25–34	167	22.0
35–44	280	36.9
45–54	158	20.8
55–64	80	10.5
≥65	17	2.2
*Mean (SD)*	40.6 (11.3)	
Sex		
Male	721	95.0
Female	38	5.0
Nationality		
Egyptian	237	31.2
Qatari	152	20.0
Syrian	77	10.1
Jordanian	73	9.6
Indian	35	4.6
Others^*^	185	24.5
Arab	659	86.8
Non-Arab	100	13.2
Marital status		
Married	648	85.4
Single	100	13.2
Divorced/widow	11	1.4
Educational level		
Primary	38	5.0
Secondary	193	25.4
College/University	528	69.6
Employment status		
Student	36	4.7
Employed	658	86.7
Unemployed	42	5.5
Retired	23	3.0
Body mass index (kg/m^2^)		
Underweight (<18.5)	9	1.2
Normal (18.5–24.9)	116	15.3
Overweight (25–29.9)	389	51.2
Obese (≥30)	245	32.3
*Mean (SD)*	29.1 (5.3)	
Presence of comorbidities		
Yes	239	31.5
No	520	68.5
Number of comorbidities (*n* = 239)		
One	151	63.2
Two or more	88	36.8
Type of comorbidities		
Diabetes mellitus	138	18.2
Hypertension	124	16.3
Asthma/COPD	57	7.5
Cardiac disease	26	3.4

*Include: Palestinian, Lebanese, Tunisian, Pakistani, Sudanese, Yemeni, Bangladeshi, Saudi, Iraqi, Filipino, Canadian, American, Bahraini, Moroccan, Omani, British.

Table 3 illustrates the smoking-related characteristics of the study population. The median (IQR) age of smoking initiation among participants was 18 years (15–20). Almost half of the participants (50.2%) reported being smokers for 10–19 years. A wide range of cigarettes smoked per day (CPD) and breath CO was reported with a median (IQR) of 24 (20–25) CPD and 16 (6–23) ppm, respectively. Regarding the type of smoking cessation therapy, almost half of the smokers (48.7%) received varenicline alone, 42.6% received NRT, and 31.8% received a combination of both. More than half of the participants (59.5%) reported being adherent to medications. The majority of the smokers (84.3%) reported being exposed to smoking among friends and 20.0% reported being exposed to smoking at home.

**Table 3:** Participants’ smoking-related characteristics recorded at baseline and six-month follow-up (*N* = 759).

Variable	Frequency	Percent (%)
Smoking-related characteristics recorded at baseline		
Age of starting smoking, *median (IQR)*	18 (15–20)	
Number of years of smoking (years)		
0–9	99	13.0
10–19	381	50.2
20 or more	279	36.8
*Median (IQR)*	18 (13–20)	
Number of cigarettes smoked per day		
1–10	102	13.4
11–20	211	27.8
21–30	323	42.6
≥31	123	16.2
*Median (IQR)*	24 (20–25)	
Carbon monoxide concentration (ppm)		
0–9	267	35.2
10–19	267	35.2
20–29	114	15.0
30 or more	111	14.6
*Median (IQR)*	16 (6–23)	
Smoking-related characteristics recorded at six-month follow-up		
Type of therapy for smoking cessation		
Nicotine Replacement Therapy (NRT)*	323	42.6
Varenicline alone	370	48.7
Combination of NRT and Varenicline	241	31.8
Medication adherence		
Always	452	59.5
Not always	307	40.5
Exposed to secondhand smoke among friends		
Yes	640	84.3
No	119	15.7
Exposed to secondhand smoke at home		
Yes	152	20.0
No	607	80.0
Number of smokers at the same home (*n* = 152)		
One	80	52.6
Two	40	26.3
Three or more	32	21.1

ppm, particle per million; *include, nicotine patches and lozenges.

### Prevalence of attempts and quit smoking

**Figure 1:**
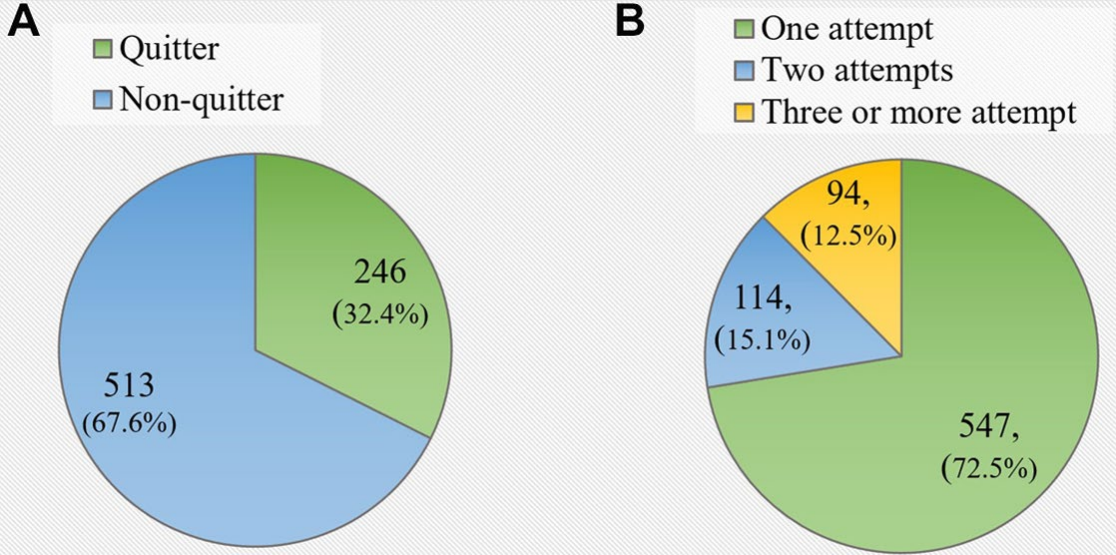
**(A)** Prevalence of 30-days quit rate among study participants at 6-months follow-up. **(B)** Number of quit attempts since the first visit to the smoking cessation clinic (*N* = 759).

The overall 30-days quit rate at the six-month follow-up interview was 32.4% (246/759) (Figure 1A). About three-quarters (72.5%) of study participants had at least one quit attempt and 12.5% had 3 or more attempts (Figure 1B).

**Figure 2:**
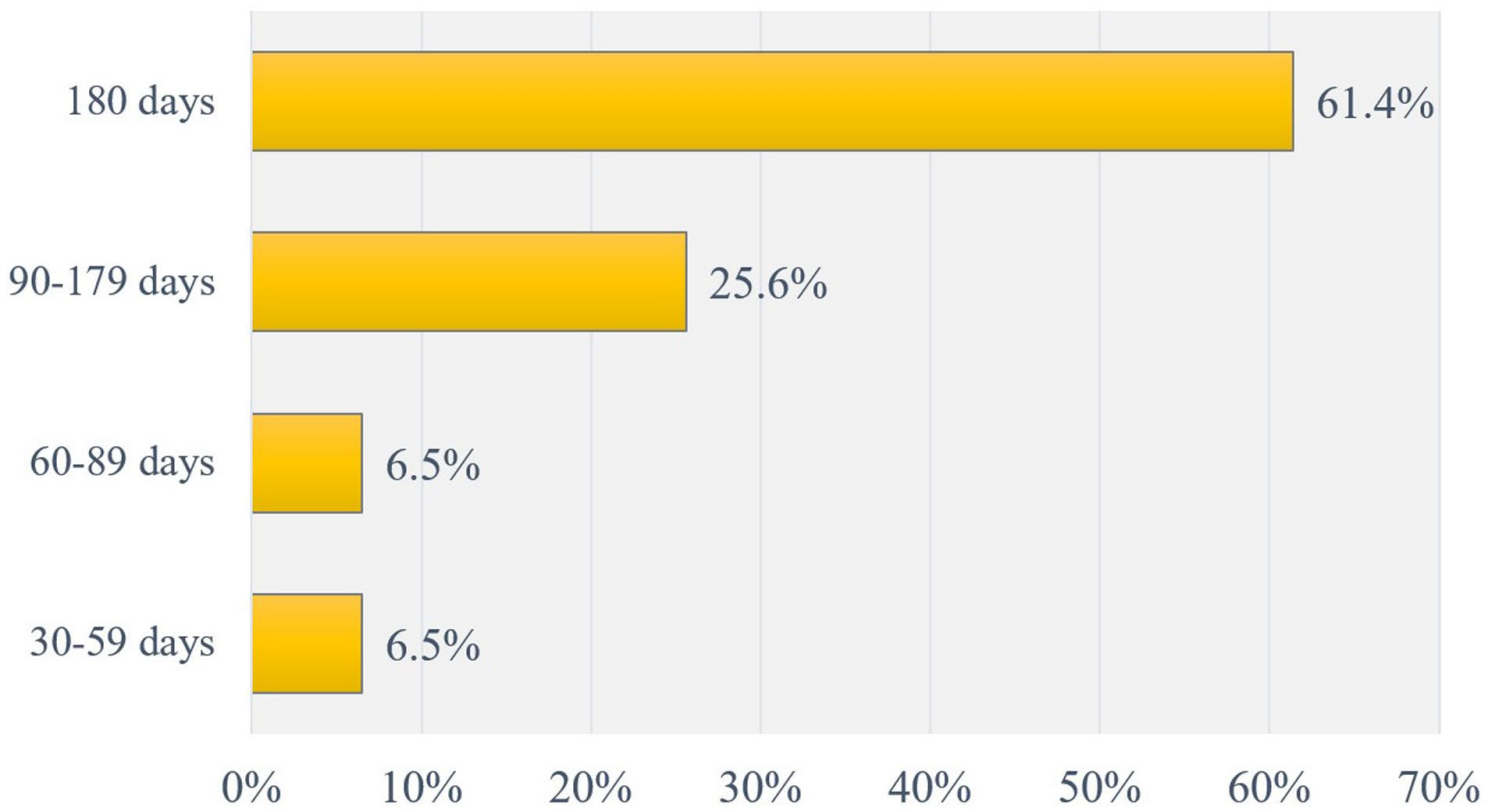
Duration of smoking abstinence among quitters at 6-month follow-up (*N* = 246).

Among participants who self-reported themselves as quitters at the six-month follow-up interview, 61.4% had been abstinent for the entire 180 days and 25.6% had been abstinent for 90 to 179 days (Figure 2).

### Factors associated with successful smoking cessation

Table 4 presents the association of the participants’ sociodemographic characteristics and their smoking behaviors with smoking cessation at 6-months follow up. Later age at smoking initiation, lower cigarette consumption at baseline, lower CO concentration at baseline, use of varenicline and/or NRT, adherence to the medication regime, having made fewer quit attempts and non-exposure to secondhand smoke among friends were factors significantly associated with successful smoking cessation.

**Table 4:** Relationship between participant’s characteristics and their smoking status (quitter vs. non-quitter) at six-month follow-up (*N* = 759).

Variable	Quitter, *n* (%)	Non-quitter, *n* (%)	Value of *p*
Age (years)			
18–24	23 (40.4)	34 (59.6)	0.250
25–44	136 (30.6)	309 (69.4)	
45–64	79 (33.2)	159 (66.8)	
65 or more	8 (47.1)	9 (52.9)	
Sex			
Female	12 (31.6)	26 (68.4)	0.901
Male	234 (32.5)	485 (67.5)	
Nationality			
Arab	206 (31.9)	452 (68.8)	0.051
Non-Arab	40 (35.7)	59 (59.0)	
Marital status			
Married	206 (31.9)	439 (68.1)	0.431
Unmarried	40 (35.7)	72 (64.3)	
Educational level			
Primary	11 (28.9)	27 (71.1)	0.822
Secondary	65 (33.9)	127 (66.1)	
College/ University	170 (32.3)	357 (67.7)	
Employment status			
Student	14 (38.9)	22 (61.1)	0.507
Employed	214 (32.6)	442 (67.4)	
Unemployed/ Retired	18 (27.7)	47 (72.3)	
Body mass index (kg/m^2^)			
Underweight (<18.5)	3 (37.5)	5 (62.5)	0.984
Normal (18.5–24.9)	37 (31.9)	79 (68.1)	
Overweight (25–29.9)	125 (32.2)	263 (67.8)	
Obese (≥30)	81 (33.1)	164 (66.9)	
Presence of any comorbidities			
Yes	77 (32.2)	162 (67.8)	0.926
No	169 (32.6)	349 (67.4)	
Age of starting smoking (years)			
At or below the age of 18	89 (29.5)	213 (70.5)	0.010*
After the age of 18	88 (40.4)	130 (59.6)	
Number of years of smoking (years)			
0–9	33 (33.3)	66 (66.7)	0.754
10–19	127 (33.5)	252 (66.5)	
20 or more	86 (30.8)	193 (69.2)	
Number of cigarettes smoked per day^a^			
1–10	44 (43.6)	57 (56.4)	0.001*
11–20	67 (31.9)	143 (68.1)	
21–30	112 (34.7)	211 (65.3)	
≥31	23 (18.7)	100 (81.3)	
Carbon monoxide concentration (ppm)^a^			
0–9	130 (49.1)	135 (50.9)	<0.001*
10–19	70 (26.2)	197 (73.8)	
20–29	25 (21.9)	89 (78.1)	
30 or more	21 (18.9)	90 (81.1)	
Used varenicline for smoking cessation			
Yes	149 (40.3)	221 (59.7)	<0.001*
No	97 (25.1)	290 (74.9)	
Used NRT for smoking cessation			
Yes	127 (39.4)	195 (60.6)	<0.001*
No	119 (27.4)	316 (72.6)	
Used combination of NRT and varenicline			
Yes	97 (40.2)	144 (59.8)	0.020*
No	149 (28.9)	367 (71.1)	
Medication adherence			
Always	179 (39.7)	272 (60.3)	<0.001*
Not always	67 (21.9)	239 (78.1)	
Number of quit attempts^b^			
One attempt	197 (36.0)	350 (64.0)	0.001*
Multiple attempts	49 (23.7)	158 (76.3)	
Expose to secondhand smoke among friends^b^			
Yes	191 (29.9)	447 (70.1)	<0.001*
No	55 (46.2)	64 (53.8)	
Expose to secondhand smoke at home^b^			
Yes	45 (29.8)	106 (70.2)	0.429
No	201 (33.2)	405 (66.8)	

*Statistically significant, ppm, particle per million; NRT, nicotine replacement therapy; ^a^at baseline; ^b^since the first visit to the smoking cessation clinic.

Table 5 illustrates the results of multivariable logistic regression analysis. Participants who started smoking after the age of 18 years were 60% more likely to quit than those who began smoking by or before the age of 18. Furthermore, participants who smoked less than 10 CPD and 11–20 CPD at baseline were more than twice as likely to quit smoking compared to those who smoked more than 30 CPD. Participants who had a lower exhaled CO level at the first visit (i.e., less than 10 ppm) were more than thrice as likely to quit compared to those who had exhaled CO level of 30 ppm or more. Smokers who used varenicline, NRT, or a combination of both for smoking cessation were more than twice as likely to quit smoking compared to those who did not use these medications. Smokers who had one quit attempt were 75% more likely to achieve successful smoking cessation at 6-month compared to those with multiple attempts. Also, participants who had no exposure to secondhand smoke among their friends were two times more likely to achieve successful quit than those with exposure.

**Table 5:** Predictors of 30-day quit rate at 6 months follow-up among study participants (Multivariable logistic regression model) (*N* = 759).

Variable	Adjusted OR (95% CI)	Value of *p*
Age (years)		
18–24	1.107 (0.453–2.706)	0.824
25–44	Reference	
45–64	0.883 (0.519–1.503)	0.647
65 or more	1.336 (0.192–9.310)	0.770
Nationality		
Arab	Reference	
Non-Arab	1.410 (0.751–2.614)	0.258
Age of smoking initiation		
At or below the age of 18 years	Reference	
After the age of 18 years	1.620 (1.002–2.630)	0.049*
Number of cigarettes smoked/day at baseline		
1–10	2.741 (1.209–6.218)	0.016*
11–20	2.122 (1.035–4.354)	0.040*
21–30	2.228 (0.972–5.105)	0.058
≥31	Reference	
Carbon monoxide concentration at baseline (ppm)		
0–9	3.365 (1.510–7.497)	0.003*
10–19	1.161 (0.499–2.701)	0.728
20–29	1.355 (0.543–3.381)	0.515
30 or more	Reference	
Used varenicline for smoking cessation		
Yes	2.244 (1.430–3.519)	<0.001*
No	Reference	
Used NRT for smoking cessation		
Yes	2.094 (1.224–3.582)	0.007*
No	Reference	
Used a combination of NRT and varenicline		
Yes	2.022 (1.007–4.057)	0.048*
No	Reference	
Number of quit attempts^a^		
One attempt	1.750 (1.204–2.544)	0.003*
Multiple attempts	Reference	
Expose to secondhand smoke among friends^a^		
Yes	Reference	
No	1.912 (1.269–2.880)	0.002*

*Statistically significant, OR, odds ratio; Cl, confidence level; ppm, particle per million; ^a^since the first visit to the smoking cessation clinic.

## Discussion

The present study assessed the rate and predictors of smoking cessation among 759 adult smokers attending smoking cessation services at PHC centers in Qatar. The 30-day quit rate at 6-month follow-up was 32.4% and indicated that factors such as low tobacco consumption, lower baseline CO concentration, smoking initiation after 18 years, use of smoking cessation pharmacotherapy, having a single quit attempt and non-exposure to secondhand smoke among friends were positively related to successful smoking cessation.

The figure obtained from this study is comparable to those reported in several public smoking cessation programs in different countries such as Malaysia (30.2%) (36), Singapore (33%) (37), Georgia-Atlanta (33%) (38), and British Columbia in Canada (37%) (39). However, lower rates were observed in Turkey (20–26%) (40) and Korea (8%) (41). Differences could be explained by several factors such as the time frame in defining quit rate, patient-related factors, and the individualized structure/content of the smoking cessation program. A number of studies have reported that older age, high educational level, being married, low number of daily smoked cigarettes, smoking initiation after the age of 20 years, as well as the absence of other partner smokers in the household are associated with an increase in the successful cessation rates (42–44).

The current study revealed that around two-thirds of the participants who managed to quit smoking at 6-month follow-up had prolonged abstinence for the whole duration of 180 days. This rate was higher compared to the percentage of long-term smoking quitters in a university-based program in South Korea (28.8%) (41), and in an outpatient smoking cessation service in Singapore (36%) (37). Higher rates in our study could be explained by the accessibility of combined therapies (e.g., NRT and varenicline) aimed to maximize benefits as well as continuous monitoring of outcomes.

Age was not found to be an influencing factor of smoking cessation in our study. Similar findings were observed in studies conducted in Hunan, China (45) and Mexico (46). However, a study on a nationally representative sample in the USA found that younger adult smokers (aged 18–24 years) were more likely to have quitted smoking for 6 months or longer than their older counterparts (35–64 years) at 8.5 and 5%, respectively. Moreover, young smokers were found to have a higher prevalence of smoke-free homes and lower levels of addiction compared to older smokers (47). In Qatar, a study among adolescents found that there was an overall increase in tobacco accessibility, availability and use between 2004 and 2013 with a decremental desire to quit smoking (48). Our study also revealed that early onset of smoking (i.e., before the age of 18 years) reduced the likelihood of successful tobacco quitting. This may point to the need for developing targeted intervention policies or programs to control the early initiation of tobacco use in youth.

In line with other studies, lower number of daily smoked cigarettes and lower exhaled carbon monoxide at baseline were considered positive predictors for successful quitting (43). Hymowitz et al. reported that heavy smokers (i.e., >25 cigarettes/day) had lower confidence and struggled to quit smoking despite their high desire (43). Such results might be explained by the effect of nicotine withdrawal symptoms associated with tobacco dependence acting as a barrier to effectively quitting smoking. Hence, more attention should be given to this category of smokers when setting up their treatment plans including intensified pharmacotherapy and close follow-up.

Another factor that showed a significant association with smoking abstinence was the use of combined NRT (e.g., nicotine lozenges and nicotine patches). In a meta-analysis of 117 trials, the risk ratio of smoking cessation using any form of NRT when compared to placebo was found to be 1.6 (95% CI 1.53–1.68) irrespective of treatment duration, setting or supplementary support (49). The effect of combining a sustained-release nicotine patch with a rapid-acting form of NRT was shown to be superior in smoking abstinence when compared to a single form of each NRT (RR 1.34, 95% CI: 1.18–1.51) (49). Additionally, our study found that treatment with varenicline, either single or combined, improved smoking cessation outcomes. This finding is consistent with a randomized controlled trial of smokers allocated to receive a 12-month duration of either varenicline, bupropion, nicotine patch, or placebo. When compared to other drugs, varenicline users had the highest continuous quit rates from week 9 to week 24, given the similar relative efficacy of all drugs (50). Furthermore, Koegelenberg et al. showed in their study that combination therapy of Varenicline and NRT was generally tolerable and more effective in achieving higher continuous abstinence rates at 12 weeks (OR 1.85, 95% CI: 1.19–2.89), 24 weeks (OR 1.98, 95% CI:1.25–3.14), and point prevalence abstinence rate at 6 months (OR 2.13, 95% CI:1.32–3.43) (51). In contrast, Hajek et at. showed that a similar intervention had failed to show a statistically significant advantage of combined varenicline and NRT when compared to varenicline alone (52). In this study, analysis of baseline participants’ characteristics by cessation treatment groups did not demonstrate significant differences. Overall, results from previous studies have yielded inconsistent findings on whether gender influences the effectiveness of NRT/varenicline and abstinence rates (53, 54). On the other hand, Ramon et al. in their randomized controlled trial found that heavy smokers were more likely to use combination therapy compared to smokers of 29 or fewer CPD (55). Differences could be related to the study samples and setting, tobacco dependency, duration of treatment, side effects of medications and presence of comorbidities (56).

In this study, smoking cessation was less likely among those who had a higher number of previous quit attempts. The aforementioned studies had shown mixed associations. Zhu SH and colleagues indicated that a higher number of past quit attempts was correlated with successful smoking cessation (57), while inverse relationship was found in other studies (58–60). It was anticipated that those with failed attempts tended to lose their self-efficacy and perceive themselves as failures halting their future quit intention. A recent failed quit attempt to stop smoking was found to be an indicator of subsequent relapse when compared to those with no recent attempt (61). Hence, motivation and intent to quit might be indicators of quit attempts but not necessarily successful ones. The role of habitual quitters who continuously try and fail was also highlighted in a prospective study (62).

Exposure to secondhand or environmental tobacco smoke between friends and families was shown to inhibit smoking cessation. Although we found a positive correlation between non-exposure to secondhand smoke among friends and successful quitting at 6 months, exposure among families was deemed an insignificant predictor. In a longitudinal study, smokers who continued to have a friend or family smoke exposure had higher odds to remain smokers in the long-term (OR 8.64, 95% CI:1.75–42.80, and OR 3.28, 95% CI:1.20–9.00 respectively) (63). The findings support the value of addressing the role of peer norms influence on smoking behavior among different cohorts. Evidence also suggests that long-term abstinence was enhanced by socioenvironmental modifications such as smoke-free policies within workplace or campus settings (64). Tackling environmental barriers at different levels should be an integral element in planning smoking prevention and control programs.

Finally, our study included mainly men of working age group with higher educational degrees and being employed. These characteristics reflect the unique structure of Qatar’s population where most of the population is male expatriates (65). Our results concord with the previously mentioned study from Bahrain (33), a member of the Gulf Cooperation Council with similar demographic features to Qatar. Both studies included mainly men with higher educational degrees in their third decade and resulted in a similar success quit rate at 6-month of follow up. In addition, the findings of this study were consistent with the predictors of successful smoking cessation reported by other Arab studies including treatment adherence, lower number of smoke packs and the use of NRT (34, 35). However, these studies were limited by a low response rate (33), or enrollment of mainly young participants with a low baseline consumption of tobacco products and carbon monoxide levels (34). We believe that our results will contribute to the understanding of smoking cessation services in the Arab countries and Gulf area in a culturally sensitive way.

### Strengths, implications on practice and research

This study has several strengths. The study was the first of its kind in Qatar to evaluate the rate and predictors of smoking cessation in smokers attending smoking cessation clinics. Another major strength of our study was the involvement of a representative sample of the population in Qatar to assess the national tobacco intervention in primary care settings. Furthermore, the use of longitudinal survey data obtained at follow-up addressed some limitations to the cross-sectional nature of the study and provided a more realistic estimate of the quit rate. Finally, the study achieved a high response rate (82.6%) despite its design.

Our results indicate that tobacco control programs should focus on younger smokers, correctively identify heavy smokers at preliminary stages by screening within clinical practice settings and the use of combined pharmacotherapy and behavioral support in their management. Concerted efforts need to be initiated to raise awareness and support smokers in their first quit attempt to avoid subsequent failures. When designing smoking interventions, psychological and social environment factors should be considered when assessing smoking intention and cessation. Environmental modifications and smoke-free laws remain key to restrict youth access to tobacco products as well as sustaining successful smoking cessation. Our results can be used to inform clinical practice as well as provide insight for further studies to more rigorously evaluate therapies and improve abstinence rates. Further research on predictors of smoking intention, cessation or failed quit attempts such as environmental changes (e.g., price increase), awareness, perception, self-efficacy, or laws are recommended to guide cost-effective tobacco control programs.

### Limitations

There are some limitations in this study. First, smoking cessation was assessed by means of self-report via follow-up telephone calls without further verification using biochemical indicators such as carbon monoxide exhale test (due to restrictions caused by the COVID-10 pandemic), potentially rendering our results less reliable. However, self-reported smoking status has been shown to have a high degree of validity (66–68). Second, we were challenged with missing data related to the Fagerstrom test due to the inconsistent collection of this variable. Third, the participants consisted mostly of middle-aged males, who were married, employed and had a high educational level, which limits the generalizability of our findings to other population groups. Finally, the study may be subjected to sampling bias due to one-fifth dropout at the follow-up interview. However, higher smoker rates were found among dropouts in other studies (69).

## Conclusion

In summary, the rate of smoking cessation at 6 months is encouraging (32.4%) and comparable to worldwide data. This study showed that low tobacco consumption, lower baseline CO concentration, smoking initiation after the age of 18, use of smoking cessation pharmacotherapy, having a single quit attempt and non-exposure to smoking among friends were predictors of successful smoking cessation. Tobacco control interventions and health education should focus on these variables: tackling younger age groups, taking into account peer influence and environmental exposure to tobacco, along with the use of combined behavioral and pharmacotherapy management, especially for heavy smokers. Interventional studies with long-term follow-up are warranted to examine enduring abstinence as well as predictors of relapses.

## Data availability statement

The original contributions presented in the study are included in the article/supplementary material, further inquiries can be directed to the corresponding author.

## Ethics statement

The studies involving human participants were reviewed and approved by the Institutional Review Board of Hamad Medical Corporation [Reference No.: MRC-01-19-324] and Primary Health Care Corporation [Reference No.: PHCC/DCR/2020/01/002]. The patients/participants provided their written informed consent to participate in this study.

## Author contributions

AA-D: conceptualization, methodology, investigation, formal analysis, funding acquisition, project administration, and writing – original draft. HM: data curation and methodology. SM: writing – review and editing. AJ: writing – original draft. WB: project administration. NS: project administration and supervision. IB: validation and supervision. All authors contributed to the article and approved the submitted version.

## Funding

This study received funding from the Medical Research Center at Hamad Medical Corporation (grant number: MRC-01-19-324). The funder was not involved in the study design, collection, analysis, interpretation of data, the writing of this article or the decision to submit it for publication.

## Conflict of interest

AA-D and AJ are employed by the Hamad Medical Corporation. HM, SM, WB, NS, and IB are employed by the Primary Health Care Corporation.

## Publisher’s note

All claims expressed in this article are solely those of the authors and do not necessarily represent those of their affiliated organizations, or those of the publisher, the editors and the reviewers. Any product that may be evaluated in this article, or claim that may be made by its manufacturer, is not guaranteed or endorsed by the publisher.
